# Tool Condition Monitoring and Remaining Useful Life Prognostic Based on a Wireless Sensor in Dry Milling Operations

**DOI:** 10.3390/s16060795

**Published:** 2016-05-31

**Authors:** Cunji Zhang, Xifan Yao, Jianming Zhang, Hong Jin

**Affiliations:** 1 School of Mechanical and Automotive Engineering, South China University of Technology, Guangzhou 510640, China; zhang.cunji@mail.scut.edu.cn (C.Z.); xtwgiqqk@163.com (J.Z.); 2 Department of Information Engineering, Guangxi College of Water Resources and Electric Power, Nanning 530023, China; 3 College of Engineering, South China Agricultural University, Guangzhou 510642, China; hjin@scau.edu.cn

**Keywords:** tool condition monitoring (TCM), remaining useful life (RUL), wireless sensor, wavelet analysis, wavelet packet transform (WPT), neuro-fuzzy network (NFN)

## Abstract

Tool breakage causes losses of surface polishing and dimensional accuracy for machined part, or possible damage to a workpiece or machine. Tool Condition Monitoring (TCM) is considerably vital in the manufacturing industry. In this paper, an indirect TCM approach is introduced with a wireless triaxial accelerometer. The vibrations in the three vertical directions (*x*, *y* and *z*) are acquired during milling operations, and the raw signals are de-noised by wavelet analysis. These features of de-noised signals are extracted in the time, frequency and time–frequency domains. The key features are selected based on Pearson’s Correlation Coefficient (PCC). The Neuro-Fuzzy Network (NFN) is adopted to predict the tool wear and Remaining Useful Life (RUL). In comparison with Back Propagation Neural Network (BPNN) and Radial Basis Function Network (RBFN), the results show that the NFN has the best performance in the prediction of tool wear and RUL.

## 1. Introduction

Traditional machining operations contain turning, milling, grinding and drilling, which are the most common operations in the manufacturing industry [1]. While the workpiece is machined, the contact between the cutter and workpiece causes the cutter shape to change, either tool wear gradually or tool breakage suddenly [2]. The problem of machine downtime continues to plague the manufacturing industry. Some sources that lead to downtime are unavoidable, for example, a workpiece is often transmitted from one workstation to another, which needs the time of dismantling and installation. In addition, a machine needs scheduled maintenance to ensure its normal operation. However, there are other downtime sources that could be avoidable, for example, the downtime caused by the tool wear or tool breakage. The failure of machine tools can attribute up to 20% of machine downtime [3]. Even if the tool does not be broken, the use of blunt tool or damaged cutter can result in extra strain on the processing equipment and quality loss in the machined workpiece. The purpose of Tool Condition Monitoring (TCM) is to adopt corresponding sensor signal processing techniques to monitor and predict the cutter state, in order to reduce the losses due to tool wear or tool damage. A powerful TCM system can improve productivity and guarantee product quality, which has a considerable influence on machining efficiency. Hence, TCM is considerably important in the manufacturing industry.

The measuring methods of tool wear are classified as direct (intermittent, offline) and indirect (continuous, online) methods based on monitoring period of signal acquisition. In direct measuring methods, such as tool-workpiece junction resistance, radioactivity, vision inspection, and optical and laser beams, the shape parameters of the cutter are measured by microscope, surface profiler, *etc.* [3]. The direct measuring methods have advantages of acquiring accurate dimension changes due to tool wear. However, these methods are vulnerable to field conditions, cutting fluid and various disturbances and are usually performed offline, and interrupts normal machining operations because of the contact between the tool and the measuring device, which severely limits the application of direct measuring methods. In indirect measuring methods, the tool wear is achieved by the corresponding sensor signals [4]. The measuring accuracy is lower than that of the direct measuring methods. However, they have the advantages of easy installation and easy to implement online in real time. This study focuses on indirect methods.

In the indirect methods, tool wear is measured based on various sensor signals containing cutting force, torque, vibration, Acoustic Emission (AE), sound, surface roughness, temperature, displacement, spindle power and current. Among these sensors, cutting force, vibration and AE measurements are robust and have been used more frequently than any other sensor measurement methods, and are more fit for the industrial field environment [5]. The features of the signals correlating to the tool wear are captured to monitor tool condition. To do this, a mass of signal processing methods were used, such as time series modeling, Fast Fourier Transform (FFT) and time–frequency analysis, the amount of calculation involved in corresponding parameters with tool wear is enormous. Wavelet Transform (WT) is a well-developed signal processing method and has been successfully used in various science and engineering fields. In the process of TCM, the sensor signals contain information and noise typically. Therefore, it is needed to de-noise and extract the features that contain the characteristics of the tool wear from various noise disturbances [6].

**Figure 1:**
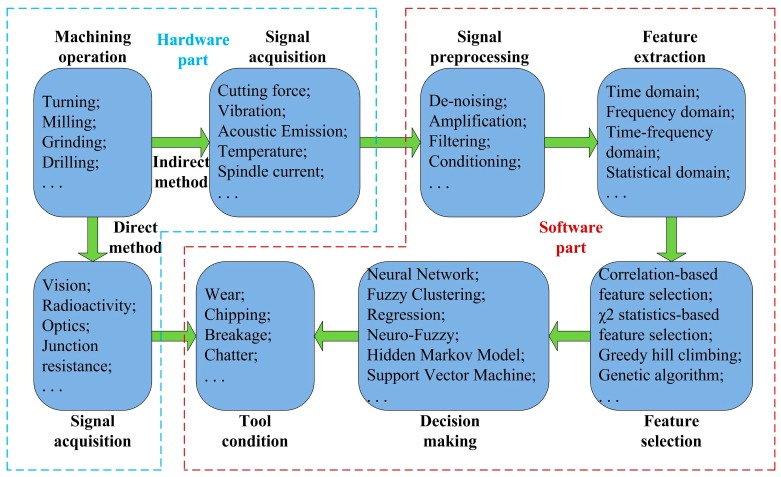
The framework of a Tool Condition Monitoring (TCM) system.

Generally, a TCM system consists of hardware and software parts to perform signal acquisition, signal preprocessing, features extraction, features selection and decision making [5]. A TCM system framework is presented in Figure 1. Signal acquisition belongs to the hardware part, but the remainder of the process including signal analysis, tool condition monitoring and Remaining Useful Life (RUL) prognostic belong to the software part. A reliable TCM system can prevent the occurrence of downtime and optimize the tool utilization in modern workpiece machining. TCM and RUL prognostics play an important role in modern manufacturing industry.

Milling operations include face milling and end milling [1]. Face milling is used for removing material from the workpiece surface by multi-toothed and rotating cutter; however, end milling is used for performing peripheral machining of a workpiece by multi-toothed and helical cutter, which is often used to remove light duty material to create the final polished surface on a workpiece. Cutters that use end milling are classified as taper, ball-nose, square-nose and cylindrical based on their geometries [3]. Especially, it is relatively long and thin and has different cutting edge geometries, and it often resembles drilling tool. End milling is often the last process in the workpiece machining and has an important impact on the quality of the final product.

In this paper, the tool conditions of a micro milling machine are monitored in real time by a wireless triaxial accelerometer. The captured vibration signals are de-noised by wavelet analysis; the features of de-noised signals are extracted in time domain, frequency and time–frequency domains [7]; features selection is performed by the criterion of correlation coefficient between the signal feature and tool wear; and three Neural Network (NN) algorithms are adopted to predict the tool wear and URL in dry milling operations.

The remainder of this paper is organized as follows: Section 2 reviews the literature of TCM and URL Prognostic in machining, states the advantages and challenges of wireless sensors, and presents problems need to be solved. Section 3 gives the theoretical framework and learning algorithm of NFN. Section 4 gives a physical experiment to complete the TCM and URL Prognostic in a micro milling machine as an example. Conclusions and suggestions for future work are given in Section 5.

## 2. Literature Review

Recently, many researchers concentrated on TCM and URL Prognostics with different types of sensors and signal processing methodologies in different machining operations. Some key findings are presented and summarized according to different type of sensors in the following.

### 2.1. Cutting Force and Torque

When a workpiece is machined, the tool becomes blunt, caused by the friction force between the cutter and the workpiece. The blunt tool requires larger cutting force to machine the same workpiece than the sharp one; that is to say, the static and dynamic cutting forces increase with the incremental tool wear, and the cutting force is an indication of tool wear in cutting condition. Torque is measured to respond to the deformation in torsional load. As such, the two types of sensing measurements are important in a reliable TCM system.

The cutting force was separated from the mixture of the Gaussian and non-Gaussian noise sources for solving the Blind Source Separation (BSS) problem in micro milling TCM [8]. The cutting force was measured to realize online monitoring of the tool condition for multi-toothed milling processes, where the dynamic characteristics of the force signal were depicted accurately and effectively [9]. Two cutting force components, *i.e.*, cutting speed (or feed rate) and cutting depth, were applied to realize online TCM during the face milling operation [10]. Cutting force signals were processed in tool wear real-time estimation [11], and three different methods were applied to extract corresponding features from the acquired signals. Jemielniak *et al.* [12] performed a comparison of signals derived from the laboratorial and industrial cutting force sensors, and the availability of the sensor outputs was researched to prove that cross talk between the channels was a significant influence for measuring the cutting force during a laboratorial simulation of industrial conditions. The approach to estimate tool wear was proposed in milling, and a lot of experiments were completed to verify the relationship between tool wear and cutting force coefficients, which include the cutting parameters containing spindle speed, cutting depth and feed rate [13]. Gao *et al.* [14] introduced a data-driven model framework for TCM in a machining operations based on the statistics of cutting forces. The orthogonal force and unidirectional strain components were compared, resulting in a probability of a difference less than 5% points between the flank wear estimation errors of the two processing strategies was at least 95% [15]. Kaya *et al.* [16] proposed an online TCM system based on the cutting force and torque signals, and a high correlation and low error ratio between the actual and predicted values of flank wear were obtained. A torque sensor was used to monitor the condition of milling cutters [17], and the efficiency and quality of the product were improved obviously.

### 2.2. Vibration

Vibration occurs due to the effect of machine components and machining process, whereas chatter results from the waviness regeneration caused by the contact between the workpiece surface and the tool at specific spindle frequencies [18]. The vibration signal reflects a tool condition, which varies with the incremental tool wear. Generally, an accelerometer is used to capture the vibration signal.

The acquired vibration signals were used to extract the time–frequency features [19], whereas many of these features containing little useful information could be discarded based on the statistical selection criteria in TCM system. A soft-computing technique was applied to estimate the tool wear with the vibration signals [20], and the prediction performance comparison of the proposed model with three other models was summarized. Yesilyurt *et al.* [21] indicated the existence and development of tool wear using vibration signals in the milling process, and the features of tool wear were revealed by the scalogram and its mean frequency. Xu *et al.* [22] proposed an energy spectrum analysis method of vibration signals for TCM, and established the estimation method for tool wear. Wang *et al.* [23] adopted vibration sensors to realize multi-category tool wear classification, and proposed the nearest neighbor based rule to perform the fast selection of v factor based on training samples, and Locality Preserving Projection (LPP) method was utilized to reduce the dimension of feature vectors by extracting the lower dimensional manifold characteristics. The vibration signals were processed by a high-speed Fast Fourier Transform (FFT) analyzer, and the effect of machining parameters on workpiece vibration, machined surface roughness and volume of metal removed was estimated in boring of steel [24]. Zhang *et al.* [25] demonstrated a TCM approach using the vibration signals acquired by a low-cost data acquisition system in an end milling operations.

### 2.3. Acoustic Emission

Acoustic Emission (AE) is defined that the transient elastic energy spontaneously release**s** in the material undergoing the deformation or fracture or both, and the energy strongly depends on the deformation rate, the strain force and the material’s volume. The primary source of AE is the sliding friction between the cutter and the workpiece [5].

The AE feature set was selected in a specific monitoring system [26], which were more efficient to estimate tool wear on various machining conditions. A micro milling type-2 fuzzy TCM system using multiple AE signal features was introduced in [27], where the URL prognostic was in accordance with the tool states during the micro milling operations. AE signal was applied in TCM [28]; the local characteristics of frequency band containing the main energy of AE signals were described in various cutting conditions. AE signals were filtered by a narrow band-pass filter, and the upper envelope for the harmonic signal was obtained by the analog hardware [29], and the encoding parameters for the envelope of those signals were classified by the Adaptive Resonance Theory (ART) and Abductor Induction Mechanism (AIM) to predict tool wear. An AE sensor was used to estimate the tool wear in milling operations of different materials including aluminum, copper and steel alloy [30], the tool wear mechanism such as adhesion and plastic deformation were indicated through the Scanning Electron Microscope (SEM) and Energy Dispersive X-ray Analyses (EDAX). AE was adopted to predict the tool wear by the constructive learning in machining operations [31], which generated compact architecture using less training time and performed well on the untrained data.

### 2.4. Other Sensors and Multi-Sensors Fusion

As is known to all, a robust and trusted TCM system needs the application of multiple meaningful signal features, which best reflect the tool wear [32]. Thus far, there are various TCM approaches, most of which often performed based on cutting force, AE and vibration signals. However, many other sensors such as current and voltage are used to realize the TCM, and so is the fusion of these sensors.

A real-time monitoring system of tool status based on the low-cost spindle motor current and voltage sensors was introduced [33], where the feature space filtering was described to obtain robust predictor online. The temperature distribution of a milling cutter in machining operations was estimated by cutting force [34], the tool wear area was modeled as an additional heat production zone, and the model was validated. A blunt tool needs larger cutting force than a sharp one, thus resulting in higher input power. The cutting force was estimated using an low-cost current sensor which was installed on the Alternating Current (AC) servomotor of a Computer Numerical Control (CNC) turning machine [35], and AE was used to identify the dresser wear to improve the effectiveness of the grinding process [36].

A TCM system based on the fusion of cutting force and electrical power sensors was proposed in face milling, and the error evaluation criteria based on the least square method was adopted to test the performance of models caused by the sensor fusion [37]. Huang *et al.* [38] proposed a signal processing approach for cutting force and vibration signals to recognize chatter, and the study showed that when the chatter occurred, cutting force increased dramatically by 61.9%–66.8% and machining surface roughness increased by 34.2%–40.5% in comparison with those of stable machining. Three perpendicular sensors of the cutting forces and vibration signals were adopted to form an online TCM system [39]. The cutting force and AE signals were applied to extract the meaningful features [40], and the correlation coefficients were used to evaluate the dependency between the signal features and the tool wear.

### 2.5. Advantages and Challenges of Wireless Sensors in Equipment Monitoring

A wireless sensor consists of sensing, data processing and communicating components, and its nature attributes include self-organization, mobility, rapid deployment, flexibility, scalability and inherent intelligent-processing capability [41]. Wireless sensors are used widely in many application fields such as military, environment, home, health, commercial and agriculture [42]. However, many variables such as temperature, pressure, humidity, workpiece conditions and machine status need be acquired in machining operations in manufacturing workshops. Recently, wireless sensors are used greatly in manufacturing industry, including industrial mobile robots, inventory management, equipment monitoring, environment monitoring, *etc*. There are some advantages using wireless sensors in health monitoring of the equipment. At first, the infrastructure of the monitoring system does not need cabling, which usually results in lower installation cost and shorter deployment time than wired solutions. Secondly, by real-time sensory data such as temperature, pressure, vibration and power from the equipment, the failure of the equipment is predicted, and the pre-emptive maintenance will be performed. At last, the scheduled maintenance of equipment based on wireless sensors can improve product quality and production efficiency, which reduces the labor cost and human errors, and prevents costly manufacturing downtime [43]. The wireless sensor networks connect with the Internet, so manufacturers can acquire the real-time status of the equipment of the workshop at anytime and anywhere.

Although wireless sensors are used in many fields, there exist such cases that cannot be solved in an efficient way, for example, data rates, tolerance, topologies, robustness, safety, security, reliability, availability, interoperability, retransmission, coexistence, energy consumption, time synchronization, self-configuration in the aspects of design for a wireless sensor [44]. From the perspective of application, the wireless sensor must be placed at the suitable position, where the measured variables can be monitored effectively and safely. For example, the TCM based on a wireless triaxial accelerometer, the sensor may be placed on different positions such as the workpiece, spindle and jig, but cannot be interfered by other things such as liquid coolant.

### 2.6. Problem Statements

Up to now, many types of sensors and signal processing techniques are used in TCM and URL Prognostic. However, most of these sensors are wired, mounted inconveniently on the machine during the machining operations, and the prognostic information is not easy to be integrated into the manufacturing system. Few researchers concentrate on wireless sensor research in TCM system. About the NFN learning algorithm, some improvements need perform to decrease the prediction error. On the basis of the above research results, this paper proposes a novel TCM method based on a wireless triaxial accelerometer in dry milling operations. Such a sensor is easy to be mounted and acquired signal under the wireless environment. The tool conditions are monitored in real time and predicted during the whole cutting process. The error back-propagation iterative algorithm and the first order gradient optimization algorithm [45] are used in NFN learning.

## 3. Theoretical Framework

Both NN and Fuzzy Logic System (FLS) [46] can be adopted to solve a problem (e.g., model prediction, pattern recognition or density estimation) separately, however, they solely do have certain advantages and disadvantages in comparison with the combination of two methods. NN is only suitable for solving a problem that is expressed by a large number of training data. At first, no empirical knowledge about the problem is required. Secondly, the comprehensible rules are not extracted directly from the NN structure. On the contrary, a FLS needs comprehensible rules instead of observed data as prior knowledge. Hence, the input and output variables needs to be described linguistically. If the linguistic rules are incomplete, incorrect or contradictory, the FLS needs to be fine-tuned, and the tuning is completed with a heuristic way. The combination of NN and FLS forms NFN, which inherits the advantages and discards the disadvantages each other.

### 3.1. The NFN Architecture

**Figure 2:**
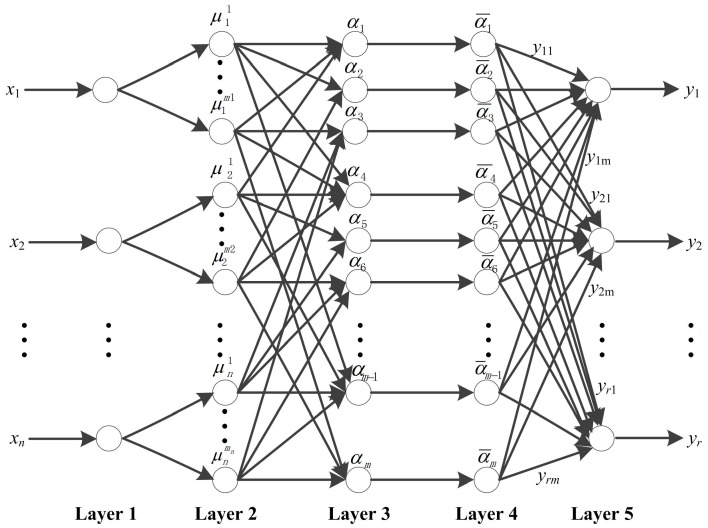
The architecture of Neuro-Fuzzy Network (NFN).

Figure 2 describes the NFN architecture [47], which is a five-layer NN based on fuzzy rules. The node at any layer *n* of the architecture has its input compared with the common NN. The node completes an operation on the input, and forms an output that is a function of its input parameter [48]. However, the connection weights and the membership functions of NFN differ greatly.

Layer 1: The node function in this layer is to transfer the input variables to next layer directly. For example, given *x* = [*x*_1_, *x*_2_, *x*_3_ …, *x_n_*]^T^, the number of nodes in this layer is *n*.

Layer 2: There are some input membership function nodes in this layer, and these nodes transform the input numerical variables into a fuzzy set (linguistic labels), that is

(1) $$\mu_{i}^{j}=\mu_{A_{i}^{j}}\left(x_{i}\right)$$

where *i* = 1, 2, 3, …, *n*, *i* is the dimension of the input variables [49]; *j* is the number of fuzzy partition for variable *x_i_*, *i.e.*, *j* = 1, 2, 3, …, *m_i_*. For example, if the Gaussian bell-shaped function is used as the membership function, then

(2) $$\mu_{i}^{j}=e^{-\frac{{\left(x_{i}-c_{ij}\right)}^{2}}{\sigma_{ij}^{2}}}$$

where *c_ij_* is the central value of the membership functions, *σ_ij_* is the width of the membership functions [50], and the number of nodes in this layer is $\sum_{i=1}^{n}m_{i}$.

Layer 3: Each node in this layer is considered the fuzzy rule, which performs the minimum–maximum fuzzy operations to the node inputs. The adaptiveness of each rule is calculated; that is

(3) $$\alpha_{j}=min\left(\mu_{1}^{i_{1}},\mu_{2}^{i_{2}},\cdots ,\mu_{n}^{i_{n}}\right)$$

or

(4) $$\alpha_{j}=\mu_{1}^{i_{1}}\mu_{2}^{i_{2}}\cdots \mu_{n}^{i_{n}}$$

where *i*_1_ ∈ {1, 2, 3, …, *m*_1_}, *i*_2_ ∈ {1, 2, 3, …, *m*_2_}, …, *i_n_* ∈ {1, 2, 3, …, *m_n_*}, *j* = 1, 2, 3, …, *m*, *m* = $\prod_{i=1}^{n}m_{i}$, and the number of the nodes in this layer is *m*.

Layer 4: The nodes in this layer perform the normalization operation; that is

(5) $${\bar{\alpha }}_{j}=\alpha_{j}/\overset{m}{\underset{i=1}{\sum }}\alpha_{i}$$

where *j* = 1, 2, 3, …, *m*, and the number of nodes in this layer is *m*.

Layer 5: The nodes in this layer perform the defuzzification and compute the outputs, each rule is

(6) $$y_{ij}=p_{j0}^{i}+p_{j1}^{i}x_{1}+\cdots +p_{jn}^{i}x_{n}=\overset{n}{\underset{l=0}{\sum }}p_{jl}^{i}x_{l}$$

where $p_{ji}^{l}$ is the connection weight coefficient, *i* = 1, 2, 3, …, *r*; *j* = 1, 2, 3, …, *m*; *l* = 0, 1, 2, 3, …, *n*, *r* is the number of output values, *x*_0_ is a constant, and *x*_0_ = 1, and the number of nodes in this layer is *m*. The outputs of NFN are

(7) $$y_{i}=\overset{m}{\underset{j=1}{\sum }}{\bar{\alpha }}_{j}y_{ij}$$

where *i* = 1, 2, 3, …, *r*, and *y_i_* is the weights sum of all the rules.

### 3.2. NFN Learning Algorithm

The learned parameters include $p_{ji}^{l}$, *c_ij_* and *σ_ij_*, which are computed and fine-tuned with the error back-propagation iterative algorithm and the first order gradient optimization algorithm. The final target of this algorithm is to minimize the error *E*, which is defined in Equation (8).

(8) $$E=\frac{1}{2}\overset{r}{\underset{i=1}{\sum }}{\left(t_{i}-y_{i}\right)}^{2}$$

where *t_i_* is the actual output value, however, *y_i_* is the predicted output value.

The connection weight coefficient $p_{ji}^{l}$ is fine-tuned as follows:

(9) $$\frac{\partial E}{\partial p_{ji}^{l}}=\frac{\partial E}{\partial y_{l}}\frac{\partial y_{l}}{\partial y_{lj}}\frac{\partial y_{lj}}{\partial p_{ji}^{l}}=-\left(t_{l}-y_{l}\right){\bar{\alpha }}_{j}x_{i}$$

(10) $$p_{ji}^{l}\left(k+1\right)=p_{ji}^{l}\left(k\right)-\beta \frac{\partial E}{\partial p_{ji}^{l}}=p_{ji}^{l}\left(k\right)+\beta \left(t_{l}-y_{l}\right){\bar{\alpha }}_{j}x_{i}$$

where *i* = 1, 2, 3, …, *n*; *j* = 1, 2, 3, …, *m*; *l* = 1, 2, 3, …, *r*, and *β* is the learning rate.

After the coefficient $p_{ji}^{l}$ is fine-tuned, it becomes a constant. The parameters of *c_ij_* and *σ_ij_* are fine-tuned as follows:

(11) $$\delta_{i}^{\left(5\right)}=t_{i}-y_{i}$$

where *i* = 1, 2, 3, …, *n*.

(12) $$\delta_{j}^{\left(4\right)}=\overset{r}{\underset{i=1}{\sum }}\delta_{i}^{\left(5\right)}y_{ij}$$

where *i* = 1, 2, 3, …, *n*; and *j* = 1, 2, 3, …, *m*.

(13) $$\delta_{j}^{\left(3\right)}=\delta_{j}^{\left(4\right)}\overset{m}{\underset{\begin{array}{c} i=1\\ i\neq j \end{array}}{\sum }}\alpha_{i}/{\left(\overset{m}{\underset{i=1}{\sum }}\alpha_{i}\right)}^{2}$$

where *i* = 1, 2, 3, …, *n*; and *j* = 1, 2, 3, …, *m*.

(14) $$\delta_{ij}^{\left(2\right)}=\overset{m}{\underset{k=1}{\sum }}\delta_{k}^{\left(3\right)}s_{ij}e^{-\frac{{\left(x_{i}-c_{ij}\right)}^{2}}{\sigma_{ij}^{2}}}$$

where *i* = 1, 2, 3, …, *n*; *j* = 1, 2, 3, …, *m_i_*; and *k* = 1, 2, 3, …, *m*. If the rule is calculated with Equation (3), then *s_ij_* = 1, else *s_ij_* = 0. If the rule is calculated with Equation (4), $s_{ij}=\prod_{\begin{array}{c} j=1\\ j\neq i \end{array}}^{n}\mu_{j}^{i}$, else *s_ij_* = 0. *c_ij_* and *σ_ij_* are fine-tuned as follows:

(15) $$\frac{\partial E}{\partial c_{ij}}=-\delta_{ij}^{\left(2\right)}\frac{2\left(x_{i}-c_{ij}\right)}{\sigma_{ij}}$$

(16) $$\frac{\partial E}{\partial \sigma_{ij}}=-\delta_{ij}^{\left(2\right)}\frac{2{\left(x_{i}-c_{ij}\right)}^{2}}{\sigma_{ij}^{3}}$$

(17) $$c_{ij}\left(k+1\right)=c_{ij}\left(k\right)-\beta \frac{\partial E}{\partial c_{ij}}$$

(18) $$\sigma_{ij}\left(k+1\right)=\sigma_{ij}\left(k\right)-\beta \frac{\partial E}{\partial \sigma_{ij}}$$

where *i* = 1, 2, 3, …, *n*; *j* = 1, 2, 3, …, *m_i_*; *k* = 1, 2, 3, …, *m*. and *β* is the learning rate. The learning process is repeated until a preset error *E* is obtained.

## 4. Experimental Results and Discussion

In this section, the indirect method for TCM and RUL prognostics is evaluated experimentally. These steps, data acquisition, data preprocessing, feature extraction, feature selection, model building and wear prediction, are performed by physical experiments.

### 4.1. Experimental Setup and Data Acquisition

**Figure 3:**
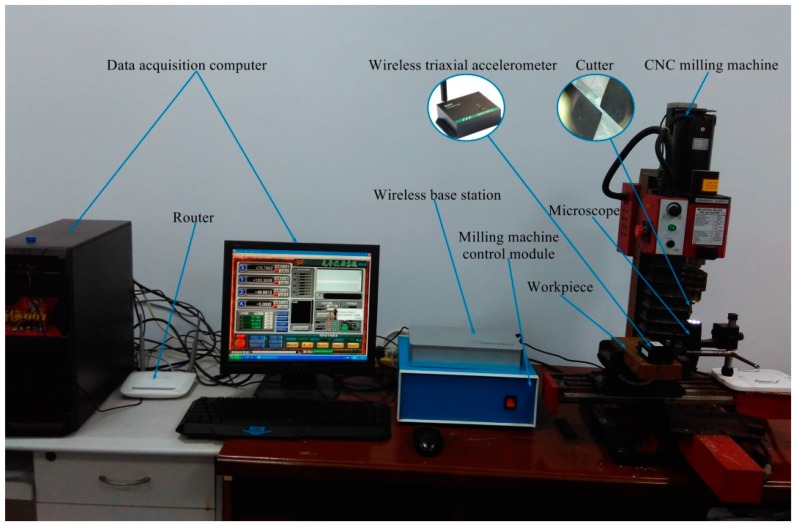
The experimental setup of TCM) and Remaining Useful Life (RUL) prognostic system.

The experimental setup for TCM and RUL prognostic is shown in Figure 3. A mini CNC milling machine (Xendoll Tech C000017) with spindle speed up to 2500 rpm/min is used in the experiment. A tempered steel (HRC52) is machined as a workpiece using micro grain carbide two-flute end milling cutter (Type: Seco S550, 6 mm diameter) coated with multilayer coatings of Titanium Aluminum Nitride (TiAlN). A wireless triaxial accelerometer (Type: M69) manufactured by Mukun Tech is mounted on the workpiece to measure the vibrations in three directions (*x*, *y* and *z*) during cutting operation. The outputs of the wireless sensor are processed by the corresponding wireless base station (Type: M90), and the vibration signals are transmitted to the upper computer through the Local Area Network (LAN).

The machining operation is carried out with feed rate 1000 mm/min, and spindle speed 2500 rpm/min. The sampling rate of the triaxial accelerometer is 1 kHz/channel. The workpiece cutting length is 50 mm in the feed direction. After completing one *x*-axis direction cutting, then the cutter returns to the starting point. The cutting depth is 0.2 mm in *z*-axis direction. In this experiment, the cutter is used to machine the groove of the workpiece. The total length of the groove (*i.e.*, 300 cuts) is 50 mm × 300 = 15,000 mm. The tool wear is measured by a handheld digital microscope (type: MSUSB401), with a high resolution camera. The combination of microscope and Anyty software is applied to acquire, measure and store images [51], and the tool wear is measured after each cut during dry milling operations.

### 4.2. Signal Preprocessing

As a very important part and a practical problem in TCM system, signal preprocessing includes the amplification, de-nosing and conditioning of the acquired raw signal. The better the raw signal is de-noised, the more accurate the tool wear is predicted. Noise always present in machining operations, especially in high precision machining [2]. Generally, noise is not concerned in TCM systems due to the small effect on the final prediction result. However, in high precision machining, the acquired sensor signal is very small, and the Signal Noise Ratio (SNR) is low. In such cases, the noise must be cleared before further analysis in the TCM system.

The signal model containing noise is expressed in Equation (19):

(19) s(t)=x(t)+σe(t)

where *s*(*t*) is the acquired raw signal, *x*(*t*) is the desired meaningful signal, *e*(*t*) is the noise, and *σ* is the standard deviation of *e*(*t*). In practice, the acquired raw signal is often a discrete time signal with equal time intervals. The signal model with noise in discrete-time is expressed in Equation (20):

(20) s(n)=x(n)+σe(n)

where *n* is the sampling serial number of the signal.

Wavelet analysis is used in signal de-noising. Wavelet localizes the signal features to different scales, and preserves important signal features while de-noising. The basic idea of wavelet de-noising is that the WT leads to a sparse representation for the raw signal. The WT concentrates on the important signal features in some large-amplitude wavelet correlation coefficients, those of which are smaller in magnitude are usually contributed by noise, so those small coefficients are shrunk or removed under the condition of without affecting the signal quality. By thresholding the coefficients, the reconstructed (de-noised) signal is obtained by Inverse Wavelet Transform (IWT).

**Figure 4:**
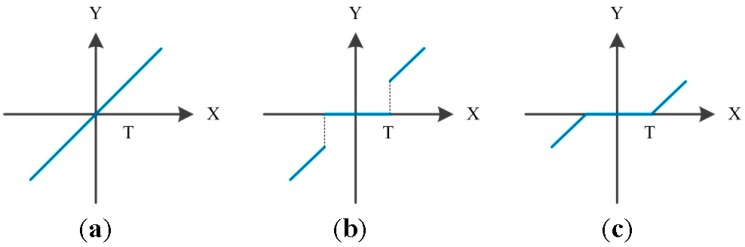
(**a**) Raw signal; (**b**) hard thresholding signal; and (**c**) soft thresholding signal.

The selection and rescaling of the threshold are crucial in wavelet de-noising. Small/large threshold values may result in over-fitting/under-fitting the signal. There are four threshold selection rules, namely, the principle of Stein’s Unbiased Risk Estimate, the heuristic threshold selection, the universal threshold, and minimum–maximum threshold selection. The threshold rescaling rules contain no rescaling, rescaling using a single estimation of level noise based on first-level coefficients, and rescaling using level-dependent estimation of level noise. The thresholding techniques containing both soft thresholding and hard thresholding are developed in [52]. Hard thresholding is expressed in Equation (21), and soft thresholding in Equation (22). The threshold handling methods are illustrated in Figure 4.

(21) $$Y=\{\begin{array}{c} X,\;\left|X\right|>T\\ 0,\;\left|X\right|\leq T \end{array}$$

(22) $$Y=\{\begin{array}{cc} sign\left(X\right)\left(\left|X\right|-T\right), & \left|X\right|>T\\ 0, & \left|X\right|\leq T \end{array}$$

**Figure 5:**
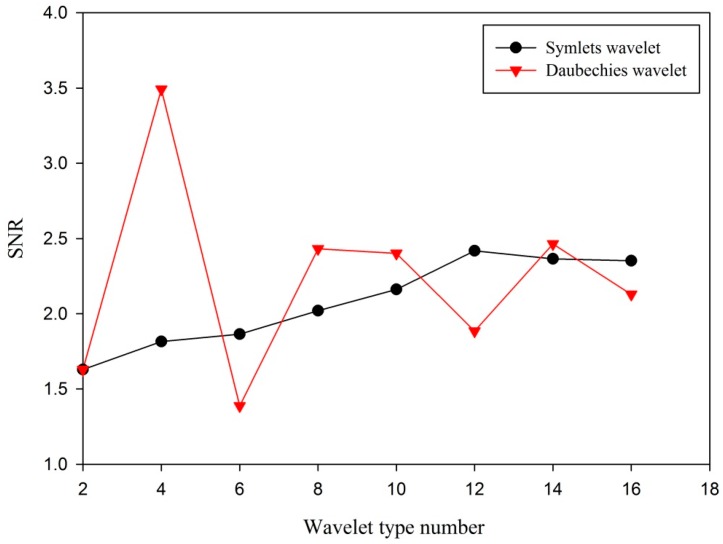
The comparison of vibration signal de-noising performance based on different wavelets.

Here, the acquired three channels of vibration signals are de-noised based on WT, and the SNR is used as a criterion to measure the performance of signal de-noising. For example, the vibration signal is decomposed at level 5 by different symlets- and daubechies-family wavelets, and the detail coefficients are rescaled using heuristic threshold selection, level-dependent estimation of level noise, and soft thresholding. The de-noised signal is reconstructed by the rescaled wavelet coefficients. The optimal wavelet de-noising parameters are selected based on the SNR criterion. The comparison of vibration signal de-noising performance based on symlets- and daubechies-family wavelets is illustrated in Figure 5.

**Figure 6:**
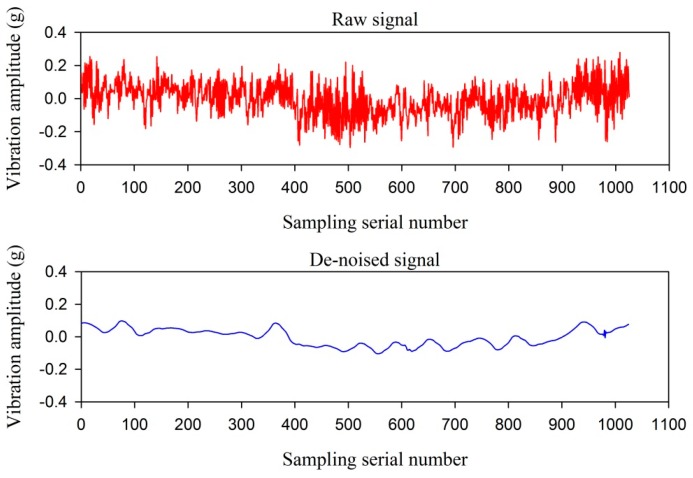
The raw vibration signal and the de-noised vibration signal.

As can be seen from Figure 5, when the vibration signal is decomposed by db4 wavelet, the de-noising performance is optimal, and the SNR is 3.49. The wavelet is selected to decompose and de-noise the vibration signal. The raw vibration signal and the de-noised vibration signal are illustrated in Figure 6.

### 4.3. Feature Extraction

The preprocessed signal is very large in volume, which is needed to further extract features. The ultimate goal of features extraction is to reduce the dimension of the original signal; meanwhile, the extracted features associate well with the cutter wear, and are not affected by process conditions. Generally, features are extracted in time, frequency, time–frequency and statistical domains [5]. The different extraction approaches have different abilities in extracting the meaningful information about tool wear.

#### 4.3.1. Feature Extraction in Time Domain

Feature extraction in time domain is often used, which considers the magnitude of the signal. The statistical method is applied to extract the time domain features such as the maximum, mean, Root Mean Square (RMS), variance, standard deviation, skewness, kurtosis, peak-to-peak and crest factor. The extracted time domain features from the vibration signal are summarized in Table 1. Time domain features are simple and only reflect the signal changes over time, and thus the frequency domain features need to be extracted too.

#### 4.3.2. Feature Extraction in Frequency Domain

**Figure 7:**
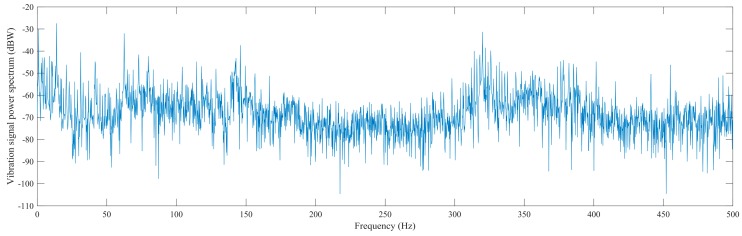
The power spectrum of the vibration signal.

Frequency domain features reflect the signal’s power distribution over a range of frequencies. Fast Fourier Transform (FFT) is used to extract the frequency domain features, while the spectrums of frequency components are the frequency domain representation of the signal. Power spectrum is measured in the 0 to half of sampling rate frequency band. Such an example for vibration signal is illustrated in Figure 7. There are a lot of extracted features, such as the maximum, sum, mean, variance, skewness, kurtosis of band power spectrum and relative spectral peak per band, as summarized in Table 2. Frequency domain features only reflect the signal changes over frequency and do not provide any type of time information, and thus it is needed to further extract the time–frequency domain features.

#### 4.3.3. Feature Extraction in Time–Frequency Domain

The Fourier Transform (FT) is usually used to analyze the spectrum of the stationary signal. However, for nonstationary signal, the Short-Time Fourier Transform (STFT) is adopted. WT is usually used to extract features in time–frequency domain, and provides localization information of a signal in time domain and frequency domain simultaneously. As such, WT is more effective in analyzing nonstationary signal than any other time–frequency approaches because of its sparsity and localization properties [5].

**Figure 8:**
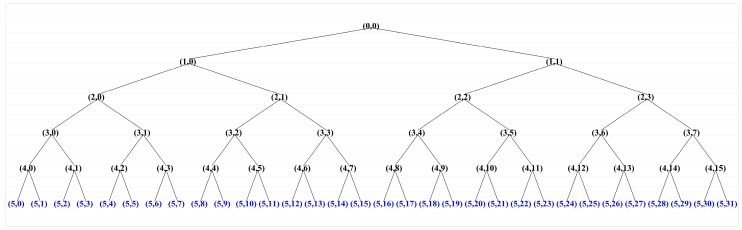
The wavelet packet tree of the vibration signal.

**Figure 9:**
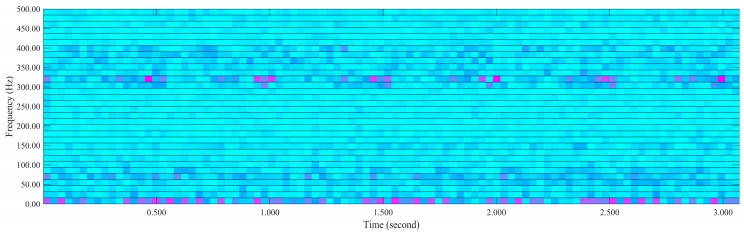
The wavelet packet spectrum of the vibration signal.

There exist Continuous Wavelet Transform (CWT) and Discrete Wavelet Transform (DWT) in time–frequency analysis [53]. However, a significant disadvantages of the CWT is a large amount of calculation, and for the DWT, the frequency resolution degree is considered very coarse in practical time–frequency analysis. As a compromise between the DWT and CWT techniques, wavelet packet provides an effective selection with sufficient frequency resolution. Wavelet Packet Transform (WPT) is applied to extract the time–frequency domain features in this paper. For example, the vibration signal features are extracted features by a five-level WPT decomposition, where 32 terminal nodes are obtained, as illustrated in Figure 8. The wavelet packet spectrum contains the absolute values of the coefficients from the frequency-ordered terminal nodes. The wavelet packet spectrum of the vibration signal in both time and frequency domains is illustrated in Figure 9. The terminal nodes provide the finest level of frequency resolution in the wavelet packet transform. The 32 wavelet packet coefficients are considered the time–frequency domain features of the vibration signal.

### 4.4. Feature Selection

There are 144 features to be extracted from three de-noised vibration signals in time, frequency and time–frequency domains. The volume of signal features is very large, many of which are much distorted, or no correlation on cutter wear. TCM and RUL prediction with all the signal features are not the best selection, because irrelevant and redundant features are able to negatively influence the performance of the monitoring and prediction model. In order to improve the accuracy of the prediction model and increase the efficiency of calculation performance of a TCM system, it is desirable that the features should be optimized according to a criterion. The feature selection technique is adopted to reduce the number of utilized features. For this end, there are a lot of techniques, for example, Correlation-based Feature Selection (CFS) method, Pearson’s chi-squared (χ2) statistics selection method, R squared (*R*^2^) statistics selection method and greedy hill climbing search algorithm [4].

Pearson’s Correlation Coefficient (PCC) [54] is adopted to select the optimal features in this paper. PCC is a measure of the linear correlation between two or more variables. When PCC is applied to measure a sample, the letter *r* is referred to as the sample Pearson correlation coefficient as defined in Equation (23):

(23) $$r=\frac{\sum_{i=1}^{n}\left(x_{i}-\bar{x}\right)\left(y_{i}-\bar{y}\right)}{\sqrt{\sum_{i=1}^{n}{\left(x_{i}-\bar{x}\right)}^{2}}\sqrt{\sum_{i=1}^{n}{\left(y_{i}-\bar{y}\right)}^{2}}}$$

where *x_i_* is one sample dataset, *y_i_* is the other sample dataset, and *n* is the sample serial number of the sample data. In this case study, *x_i_* denotes the selected dataset, and *y_i_* represents the corresponding cutter wear dataset.

The *r* value is considered the criterion of feature selection [55]. The *r* value may be negative, and the absolute value of *r* is considered to be the score of the feature correlation. A confidence level (*r* = 0.90) is set to get rid of low *r* value, and only *r* values above the level are retained in the final features. Features selected in three domains are summarized in Table 3. In all 144 signal features, 13 features are automatically selected as useful.

### 4.5. Modeling for Cutting Tool

Based on the selected features to estimate the cutter wear, a decision can be made through Artificial Intelligence (AI) approaches such as NN, FLS, NFN, HMM and SVM. NN is considered a popular choice because of its advantages of high fault tolerance, noise suppression capability and adaptiveness [5]. NFN is used to perform TCM and RUL prediction in this case study.

A total of 86,400 sets of feature data of cutter c1 and cutter c2 are generated from raw signals, and the selected feature data are 7800 sets, which are used to train rules. The data samples are normalized for further uses.

The NFN model performs from feeding the training dataset to the network from layer 1, as illustrated in Figure 2. The 13 selected features as the input variables are transmitted into the NFN. In layer 2, the numerical data are fuzzified by the input membership functions, which are the generalized bell-shaped functions. In layer 3, the initial Fuzzy Inference System (FIS) structure is generated from training data using subtractive clustering. Then the NFN is trained and learned through fine-tuning the learned parameters with the error back-propagation iterative algorithm and the first order gradient optimization algorithm. The generated rules are normalized in layer 4, and the antecedent parts of each rule are formed. In layer 5, the output is defuzzified, and the weighted global output of the NFN is calculated.

**Figure 10:**
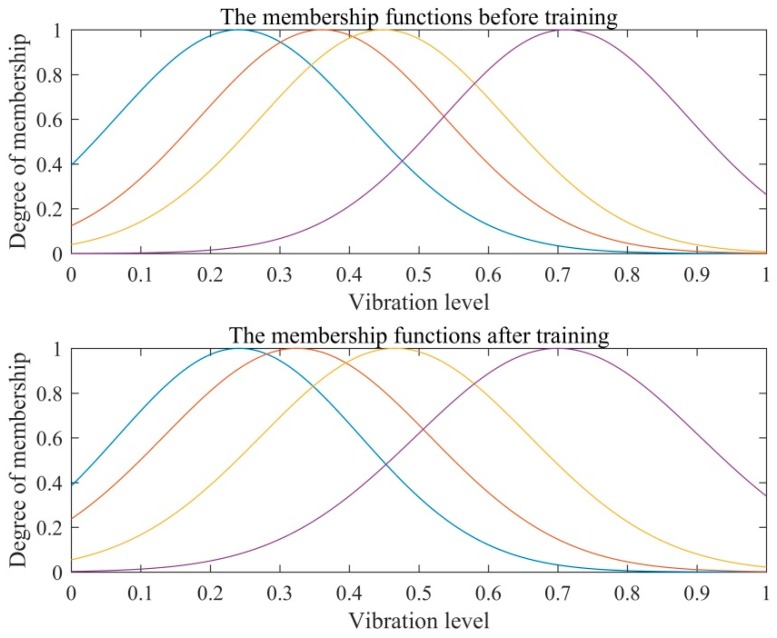
The membership function distributions of before and after the training.

Figure 10 shows the membership function distributions of the first selected feature before and after the training. The normalization of the input feature data is in *x*-axis direction, and the *y*-axis represents the membership degree. The number of the membership functions for each input variable is 4. The data sets are aggregated according to its similarity with the fine-tuning [56]. After the membership functions are constructed, the fuzzy rules are performed using fuzzy min-max operations. The total number of rules is calculated by *N*(*x*_1_)·*N*(*x*_2_)·…·*N*(*x_n_*), where *N*(*x_n_*) is the number of membership functions of the *n*th input variable.

### 4.6. Prediction of Tool Wear and RUL

#### 4.6.1. Prediction of Tool Wear and RUL Based NFN

After the NFN is trained, the test data is fed to the networks to predict the tool wear. For a new input data set, when the new fuzzy rule does not match any of the existing rules, the new rule will be replaced by a closest rule, which will inevitably transmit errors into the final results. Therefore, it is very important that a large amount of data is used to train the networks to ensure that the trained rules are comprehensive. The NFN can be retrained whenever the new dataset become available. In this case study, the 3900 feature data sets of cutter c3 and cutter c4 are fed to the NFN as test data in turn.

**Figure 11:**
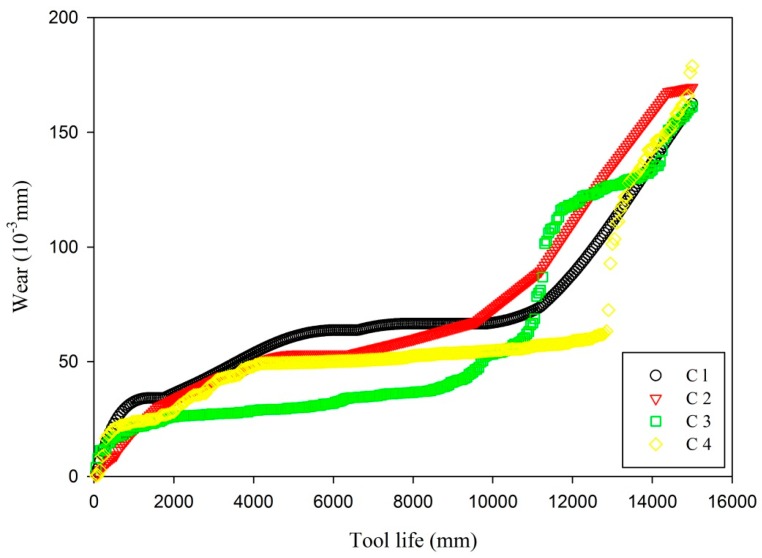
The correlation of the tool life and the tool wear.

The radii parameter of NFN is set as 0.5, and the initial FIS structure generates using subtractive clustering (genfis2). After the NFN is trained, the tool wear of cutter c3 and cutter c4 is predicted. The RUL prediction of a cutter is the ultimate aim of the TCM during the machining process. The tool life is represented using the distance that a milling cutter machines the workpiece. The correlation of the tool life and the tool wear is illustrated in Figure 11.

**Figure 12:**
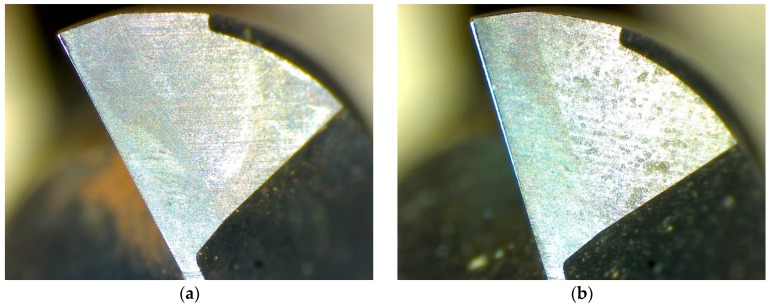
(**a**) Tool wear at cut distance 500 mm; and (**b**) tool wear at cut distance 2500 mm.

Figure 11 gives the relationship between the tool life and the cutter wear for four cutters, where the tool wear of cutter c1 and cutter c2 is measured by a handheld digital microscope, and tool wear of cutter c3 and cutter c4 is predicted with NFN. The tool wear at two cut distance for cutter c1 is shown in Figure 12, which is magnified 135 times. Initial wear of all cutters is 0; that is, the workpiece is machined by the new cutter. The figure shows that cut distance of 11,300 mm, with predicted tool wear of 101.5135 (10^−3^ mm) for cutter c3, cut distance of 12,900 mm and 12,950 mm, with predicted tool wear of 72.4967 (10^−3^ mm) and 92.9041 (10^−3^ mm) for cutter c4, respectively, where the tool wear changes significantly.

#### 4.6.2. User Interface of Tool Wear and RUL

**Figure 13:**
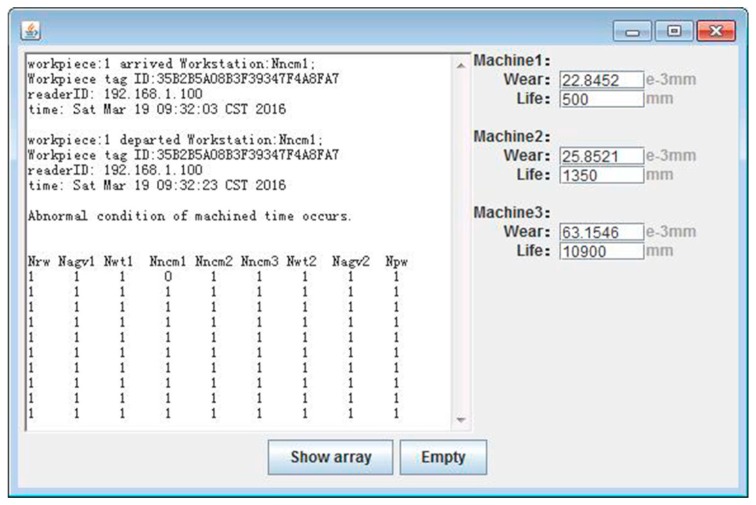
The user interface of tool wear and RUL.

A user-friendly interface of cutter wear and RUL is constructed by Eclipse and Matlab in a Windows 7 (32 bit) operating system. The workpieces are machined in the same operation environment by the same type CNC machines, equipped with the same type cutters and in the same machining conditions. Machine 1 is equipped with cutter c1, and so on. The user interface of tool wear and RUL is shown in Figure 13. The left part of this figure is the abnormal condition monitoring of workpieces by Radio Frequency Identification (RFID), which is previous work introduced in [57]. The tool wear and tool life (cut distance) of the cutters are shown in the right part of this figure.

### 4.7. Comparison with BPNN, RBFN and NFN

The same datasets are used to estimate the cutter wear with BPNN and RBFN. The parameter settings of the BPNN, RBFN and NFN are summarized in Table 4. The NFN model comprises five layers which are illustrated in Figure 2. The training function used for the BPNN is Levenberg–Marquardt, the learning algorithm for the NFN is the error back-propagation iterative and the first order gradient optimization algorithm.

The prediction performance used for three models is gauged with Mean Squared Error (MSE), Mean Absolute Percentage Error (MAPE) and *R*^2^ values. The error comparison with BPNN, RBFN and NFN is summarized in Table 5. According to the comparison with the experiment results, it can be observed that The NFN performs the best with the smallest MSE and MAPE and the biggest R^2^, and the RBFN performs the worst.

## 5. Conclusions and Future Work

In this paper, a novel approach for TCM and URL prognostics based on a wireless triaxial accelerometer is presented. The wireless triaxial accelerometer is used to detect the vibrations in three perpendicular directions (*x*, *y* and *z*) during cutting operations. The raw vibration signals are preprocessed by wavelet analysis. Different methods are applied to extract and select features. NFN is used to predict the tool wear and URL, and the NFN outperforms the others through the comparison. This prognostic approach is easy to realize and provides a basis for proactive job shop scheduling through integrating the prognostic information into the manufacturing system.

However, this study only focuses on TCM and URL prognostics based on one sensor with NFN in dry milling operations. In future work, multiple sensors will be used to measure the vibrations at different positions, such as the spindle and the jig. The information fusion of multiple sensors will be applied to predict the tool wear more accurately. At the same time, we also plan to study TCM and URL prognostics based on deep learning.

## Figures and Tables

**Table 1:** Features extracted in time domain.

Index	Feature	Description
1	Maximum	*X_MAX_* = *max*(*x_i_*)
2	Mean	*μ* = $\frac{1}{n}\sum_{i=1}^{n}x_{i}$
3	Root mean square	*X_RMS_* = $\sqrt{\frac{1}{n}\sum_{i=1}^{n}x_{i}^{2}}$
4	Variance	*X_V_* = $\frac{\sum_{i=1}^{n}{\left(x_{i}-\mu \right)}^{2}}{n-1}$
5	Standard deviation	*σ* = $\sqrt{\frac{\sum_{i=1}^{n}{\left(x_{i}-\mu \right)}^{2}}{n-1}}$
6	Skewness	*X_S_* = $\frac{1}{n}\frac{\sum_{i=1}^{n}{\left(x_{i}-\mu \right)}^{3}}{\sigma^{3}}$
7	Kurtosis	*X_K_* = $\frac{1}{n}\frac{\sum_{i=1}^{n}{\left(x_{i}-\mu \right)}^{4}}{\sigma^{4}}$
8	Peak-to-peak	*X_P2P_* = *max*(*x_i_*)-*min*(*x_i_*)
9	Crest factor	*X_CF_* = $\frac{max\left(xi\right)}{\sqrt{\frac{1}{n}\sum_{i=1}^{n}x_{i}^{2}}}$

**Table 2:** Features extracted in frequency domain.

Index	Feature	Description
1	Maximum of band power spectrum	*S_MAX_* = *max*($S{\left(f\right)}_{i}$)
2	Sum of band power spectrum	*S_SBP_* = $\sum_{i=1}^{n}S{\left(f\right)}_{i}$
3	Mean of band power spectrum	*S_μ_* = $\frac{1}{n}\sum_{i=1}^{n}S{\left(f\right)}_{i}$
4	Variance of band power spectrum	*S_V_* = $\frac{\sum_{i=1}^{n}{\left(S{\left(f\right)}_{i}-S_{\mu }\right)}^{2}}{n-1}$
5	Skewness of band power spectrum	*S_S_* = $\frac{1}{n}\frac{\sum_{i=1}^{n}{\left(S{\left(f\right)}_{i}-S_{\mu }\right)}^{3}}{S_{V}{}^{3/2}}$
6	Kurtosis of band power spectrum	*S_K_* = $\frac{1}{n}\frac{\sum_{i=1}^{n}{\left(S{\left(f\right)}_{i}-S_{\mu }\right)}^{4}}{S_{V}{}^{4/2}}$
7	Relative spectral peak per band	*S_RSPPB_* = $\frac{max\left(S{\left(f\right)}_{i}\right)}{\frac{1}{n}\sum_{i=1}^{n}S{\left(f\right)}_{i}}$

**Table 3:** Features selected for vibration signals in three domains.

Sensor	Time Domain	Frequency Domain	Time–Frequency Domain	Total	Selected Features
Vibration	27	21	96	144	13

**Table 4:** The parameter settings of the Back Propagation Neural Network (BPNN), Radial Basis Function Network (RBFN) and NFN.

Parameters	BPNN	RBFN	NFN
Learning rate	0.1	0.1	0.1
Training function (Learning algorithm)	Levenberg–Marquardt	-	Back-propagation iterative and gradient optimization
Number of layers	3	3	5
Number of nodes each layer	13, 50, 1	13, 300, 1	13, 52, 128, 128, 1
Number of samples	7, 800	7, 800	7, 800

**Table 5:** Error comparison with BPNN, RBFN and NFN.

Error	BPNN	RBFN	NFN
MSE	5.9975 × 10^−4^	1.06 × 10^−2^	3.257 × 10^−4^
MAPE	0.0308	0.1461	0.0224
*R*^2^	0.9851	0.7373	0.9919

## Supplementary Materials

Supplementary File 1
